# Healthcare staff mental health trajectories during the COVID-19 pandemic: findings from the COVID-19 Staff Wellbeing Survey

**DOI:** 10.1192/bjo.2023.497

**Published:** 2023-06-22

**Authors:** Julie-Ann Jordan, Ciaran Shannon, Dympna Browne, Emma Carroll, Jennifer Maguire, Keith Kerrigan, Sinead Hannan, Thomas McCarthy, Mark A. Tully, Ciaran Mulholland, Kevin F. W. Dyer

**Affiliations:** PhD, IMPACT Research Centre, Northern Health and Social Care Trust, Northern Ireland, UK; DClinPsych, IMPACT Research Centre, Northern Health and Social Care Trust, Northern Ireland, UK; PhD, Belfast Health and Social Care Trust, Northern Ireland, UK; DClinPsych, Northern Health and Social Care Trust, Northern Ireland, UK; DClinPsych, PhD, South Eastern Health and Social Care Trust, Northern Ireland, UK; PhD, Northern Health and Social Care Trust, Northern Ireland, UK; DClinPsych, Southern Health and Social Care Trust, Northern Ireland, UK; DClinPsych, Western Health and Social Care Trust, Northern Ireland, UK; PhD, School of Medicine, Ulster University, Northern Ireland, UK; MD, IMPACT Research Centre, Northern Health and Social Care Trust, Northern Ireland, UK; DClinPsych, PhD, IMPACT Research Centre, Northern Health and Social Care Trust, Northern Ireland, UK

**Keywords:** Depressive disorders, trauma, anxiety disorders, longitudinal, healthcare workers

## Abstract

**Background:**

Cross-sectional studies have shown that the COVID-19 pandemic has had a significant impact on the mental health of healthcare staff. However, it is less well understood how working over the long term in successive COVID-19 waves affects staff well-being.

**Aims:**

To identify subpopulations within the health and social care staff workforce with differentiated trajectories of mental health symptoms during phases of the COVID-19 pandemic.

**Method:**

The COVID-19 Staff Wellbeing Survey assessed health and social care staff well-being within an area of the UK at four time points, separated by 3-month intervals, spanning November 2020 to August 2021.

**Results:**

Growth mixture models were performed on the depression, anxiety and post-traumatic stress disorder longitudinal data. Two class solutions provided the best fit for all models. The vast majority of the workforce were best represented by the low-symptom class trajectory, where by symptoms were consistently below the clinical cut-off for moderate-to-severe symptoms. A sizable minority (13–16%) were categorised as being in the high-symptom class, a group who had symptom levels in the moderate-to-severe range throughout the peaks and troughs of the pandemic. In the depression, anxiety and post-traumatic stress disorder models, the high-symptom class perceived communication from their organisation to be less effective than the low-symptom class.

**Conclusions:**

This research identified a group of health service staff who reported persistently high mental health symptoms during the pandemic. This group of staff may well have particular needs in terms of the provision of well-being support services.

The COVID-19 pandemic has resulted in health and social care staff being exposed to an unprecedented number of primary (i.e. occupation-related) and secondary (i.e. interaction between personal- and occupation-related) work stressors. These include potentially enhanced exposure of staff and their families to COVID-19 infection, intensive work pressure and demands, increased daily experience of patient suffering and mortality, and reduced ability to engage in normal self-care activities because of workplace restrictions.^1,2^ Further, the nature of the pandemic coupled with stretched healthcare resources have made working in healthcare settings conducive to engendering experiences of ‘moral injury’, a source of psychological distress that can occur when staff have to take action or inaction they view as being incompatible with their moral and ethical code.^3^ Such experiences can be varied and range across a continuum of severity, extending from staff providing virtual consultations rather than face-to-face contacts with patients, to staff having to restrict family visiting rights for terminally ill patients.^4,5^

The research base supports the above assertions, providing robust evidence of how COVID-19 stressors have specifically affected the frequency and severity of mental health symptoms experienced by healthcare workers. High rates of moderate-to-severe depression (15%), anxiety (12%) and, in particular, post-traumatic stress disorder (PTSD) (35%) were evident among nursing and medical staff when COVID-19 first emerged in the Wuhan area of China.^6^ Estimates of ‘probable’ mental health diagnoses were generally higher among UK healthcare staff during the first wave of COVID-19 (depression 27%, anxiety 23%, PTSD 30%),^7^ with some occupations more vulnerable than others (e.g. administrative staff, nurses).^8,9^ However, although such studies provide a useful snapshot of the impact of the COVID-19 pandemic on healthcare staff at specific points in time, they have a number of methodological limitations (e.g. cross-sectional design, low response rates). Moreover, theoretical models of post-traumatic and occupational stress would posit that cumulative, complex and long-term exposure to multiple forms of ‘organisational trauma’ (e.g. restricted care provision, exposure to death and dying)^10,11^ are likely to elicit a more complicated and enduring symptom profile, including delayed-onset burnout and reduced performance.^12–14^ To maximise the effectiveness of staff well-being support strategies, it is therefore essential to monitor the persistence of mental health symptoms among staff over time and gauge relevant mitigating factors.

The COVID-19 Staff Wellbeing Survey Project is a longitudinal evaluation of mental health symptoms experienced by healthcare staff in Northern Ireland during and after the pandemic.^9^ The first two time points of the survey (November 2020 and February 2021) coincided with the most intense phase of COVID-19-related hospital activity in the UK; specifically, the second and third waves of the COVID-19 pandemic. During this period, high rates of moderate-to-severe symptoms were found for depression (30–36%), anxiety (26–27%), post-traumatic stress (30–32%) and insomnia (27–28%). A longitudinal subsample formed from staff respondents who completed the survey at multiple time points revealed that high levels of psychological distress were maintained across the waves, and were significantly affected by specific organisational risk factors (e.g. less effective communication). This was in keeping with general research on the importance of communication on staff well-being.^15^

## Subpopulations of COVID-19-related psychological symptoms

Although generic examination of current and longitudinal effects of COVID-19 stressors and moderating organisational factors are important avenues of study, an obvious criticism is that they do not scrutinise at-risk subgroups within the broader healthcare population. It is entirely possible that the reactions of healthcare staff to COVID-19 stressors are not uniform. Subpopulations likely exist within the healthcare workforce who differ in terms of how the COVID-19 pandemic affects their long-term mental health. This latent class hypothesis has been supported in studies of the general population in relation to COVID-19. McPherson et al^16^ assessed anxiety and depression levels across the first wave of COVID-19 in the UK, and revealed four subpopulations differentiated both in terms of the stability and severity of their symptoms. The majority of the sample had low levels of anxiety and depression throughout the pandemic, demonstrating considerable resilience to the demands of lockdown. However, a concerning subpopulation was the high-stable group (12% in depression and anxiety models), who had consistently high levels during the period of study. A further two groups were identified, one that started with low levels that steadily increased over time, and another group showing the reverse trend of starting high and eventually dropping down to healthy levels.

## Aims

The present study aimed to build upon the work of Jordan et al. by extending the COVID-19 Staff Wellbeing Survey to measure levels of depression, anxiety, post-traumatic stress and insomnia at two further time points: (a) when COVID-19 hospital activity in the UK was very low (May 2021) and (b) during a relatively smaller subsequent surge of COVID-19 admissions (August 2021). Growth mixture modelling was applied to the overall longitudinal data-set comprising four time points. This allowed for the examination of the possibility that sub-populations may exist within the health and social care staff workforce, each with qualitatively distinct mental health symptom trajectories.^17^ A further aim was to gain better understanding of who is at risk of long-term moderate-to-severe psychological distress and to determine how best to support them by examining personal and demographic, organisational and support-related predictors of class membership.

## Method

### Participants and design

The COVID-19 Staff Wellbeing Survey was open to all staff employed by a health and social care employer in Northern Ireland. The longitudinal survey ran at four time points separated by 3-month intervals: time point 1 (9–22 November 2020, *n* = 3834), time point 2 (8–28 February 2021, *n* = 2898), time point 3 (10–30 May 2021, *n* = 2480) and time point 4 (9–29 August 2021, *n* = 2119). Regarding COVID-19 hospital admissions in Northern Ireland, the time points coincided with a large peak in admissions (time points 1 and 2), followed by a period when admissions were very low (time point 3) and then a relatively smaller surge in admissions at time point 4.^18^ The recruitment strategy for the survey included broadcast emails to staff, adverts via staff social media, laminated posters in staff areas and screensavers. The demographic profiles of the samples at time points 1–4 are shown in Supplementary Table 1, available at https://doi.org/10.1192/bjo.2023.497. At each time point, staff were given the option of providing a work or personal email address that could be used to link their responses over time, allowing a longitudinal data-set to be formed with email address as the unique identifier. The analysis reported here is based on a sample of 585 participants whose responses could be linked for at least three survey time points. Although the age and gender profile of the longitudinal sample was comparable to that of those with responses for time point 1 only, those from nursing and midwifery, social services or other roles were more likely to drop out over time (Table 1)

**Table 1:** Demographic characteristics of participants from time point 1 only, and longitudinal participants

		Score		
Variable	Statistics	Time point 1 only	Longitudinal	Statistical comparison
Age (time point 1 only *n* = 3365, longitudinal *n* = 585), years	Mean (s.d.)	43.56 (10.54)	44.56 (10.67)	*t* (3820) = −0.76, *P* = 0.967
Gender (time point 1 only *n* = 3369, longitudinal *n* = 584)				
Male	Frequency (%)	589 (17%)	102 (17%)	*χ*^2^(1) = 0.00, *P* = 0.992
Female	Frequency (%)	2780 (83%)	482 (83%)	
Occupation (time point 1 only *n* = 3377, longitudinal n = 585)				
Nursing and midwifery	Frequency (%)	824 (24%)	100 (17%)	*χ*^2^(5) = 42.02, *P* < 0.001
Administrative and clerical	Frequency (%)	903 (27%)	195 (33%)	
Medical	Frequency (%)	212 (6%)	36 (6%)	
Professional and technical	Frequency (%)	651 (19%)	155 (26%)	
Social services	Frequency (%)	497 (15%)	68 (12%)	
Other	Frequency (%)	290 (9%)	31 (5%)	

For the longitudinal sample, time point 2 data for gender and occupation were used if time point 1 data were missing.

### Measures

#### Longitudinal mental health measures (time points 1–4)

The Patient Health Questionnaire-9 (PHQ-9)^19^ provided a measure of depressive symptoms in the previous 2 weeks. The PHQ-9 comprises nine items assessing presence and frequency of symptoms, and response is given as a four-point Likert scale (0 being not at all, 3 being nearly every day); the scale range is 0–27, with higher scores being indicative of greater levels of depressive symptoms.

The Generalised Anxiety Disorder-7 screener (GAD-7)^20^ was used to measure anxiety symptoms within the previous 2 weeks. The GAD-7 comprises seven items assessing presence and frequency of symptoms, and responses are measured with a four-point Likert scale (0 being not at all, 3 being nearly every day); the scale range is 0–21, with higher scores reflecting greater anxiety levels.

Post-traumatic stress relating to the COVID-19 outbreak in the past 7 days was assessed via the 22-item Impact of Event Scale-Revised (IES-R).^21^ This DSM-IV-compatible measure uses a five-point Likert scale to assess the presence and severity of PTSD symptoms in the past 7 days (0 being not at all, 4 being extremely; scale range 0–88).

The Insomnia Severity Index (ISI)^22^ provided a measure of past month insomnia symptoms. The ISI comprises seven items measured using a five-point Likert scale, with higher scores suggestive of greater insomnia symptoms (scale range 0–28).

Analyses on the longitudinal sample revealed high internal consistency (Cronbach's alpha) across time for depression (0.89–0.90), anxiety (0.92–0.93), PTSD (0.95–0.96) and insomnia (0.90–0.91). The established cut-off points for moderate-to-severe symptoms on these measures are ≥10 for the GAD-7 and PHQ-9, ≥26 for the IES-R and ≥15 for the ISI.^6,19,20,21,22^

#### Predictor variables (time point 1)

Personal, demographic, occupational and support variables recorded at time point 1 were used as predictors of anxiety, depression and post-traumatic stress class membership in the growth mixture models.

Demographic and personal variables included gender (male/female), age (years), occupation (administrative and clerical, medical, professional and technical, social services, other) and a measure of COVID-19 exposure (scale range 0–7, with higher scores indicating greater exposure). In addition, participants were asked to indicate if they had at least one of ten COVID-19 risk factors (coded 0 for no and 1 for yes) specified in the COVID-19 Pandemic Mental Health Questionnaire (e.g. diabetes, chronic liver disease).^23^

Occupational variables included whether they had managed patients with COVID-19 (coded 0 for no and 1 for yes); if they had been asked to consider a redeployment opportunity during the pandemic (coded 0 for no and 1 for yes); and how effective they considered communication from their organisation to be on COVID-19-related matters. Communication effectiveness was measured on a Likert scale ranging from 0 (not effective) to 4 (very effective). Further details on these demographic and occupational measures are reported in Jordan et al.^9^ Measures of supports available and support used were also included as predictors in the growth mixture models. Respondents were shown lists and asked to indicate which team supports (e.g. Schwartz rounds, ‘buddy’ system) were available to them and which staff well-being supports (e.g. staff well-being helpline, drop-in centre) they had used during the pandemic. Four predictors were formed from these data: if any team supports were available (coded 0 for no and 1 for yes), if they had used any staff supports (coded 0 for no and 1 for yes), total number of team supports available (0–8) and total number of staff supports used (0–9).

### Procedure

The survey was voluntary and completed online through the Survey Mechanics platform (Instant Insight, Ticehurst, UK; see https://surveymechanics.com). Participants indicated their consent to participate by clicking to start the questionnaire after reading the information sheet at the start of the survey. Respondents wishing to withdraw could do so by exiting the survey. Responses were only included in the data-set if respondents clicked submit at the end of the survey. The authors assert that all procedures contributing to this work comply with the ethical standards of the relevant national and institutional committees on human experimentation and with the Helsinki Declaration of 1975, as revised in 2008. All procedures involving human patients were approved by West of Scotland Research Ethics Service (reference 20/WS/0122, 26 August 2020). Participants were provided with details of psychological well-being support contacts at the start and end of the survey.

### Statistical analysis

Statistical analyses were conducted with Mplus version 8.6 (Muthén & Muthén, Los Angeles, USA; see https://www.statmodel.com).^24^ The missing data rate for the sample for all measures across time was 22% for time point 1, 10% for time point 2, 12% for time point 3 and 27% for time point 4. The first analysis step involved fitting unconditional growth models to the depression, anxiety, post-traumatic stress and insomnia data for time points 1–4. A variety of fit indices were used to determine if linear, quadratic or free time scores models provided the best fit for the models. The fit indices considered included chi-squared (*χ*^2^), comparative fit index, Tucker–Lewis index, root mean square error of approximation and standardised root mean square residual. Missing values in the growth models were dealt with via full information maximum likelihood estimation. Significant variation was evident for the slope parameters of the depression and post-traumatic stress models, but not for the insomnia model. In the anxiety model, the slope variation was non-significant and therefore fixed at zero; nevertheless, there was significant variation in the quadratic component of this model. Only the depression, anxiety and post-traumatic stress data were taken forward to the growth mixture model stages of the analysis, as the significant slope or quadratic variation suggested the presence of subpopulations in the data that varied in terms of the gradient of their mental health symptom trajectories throughout the pandemic.

In the second stage, unconditional growth mixture models were used to compare models with two to five classes for depression, anxiety and post-traumatic stress. These models were specified with variances and covariances constrained to be equal across classes (invariant approach), and with variances and covariances freed to be estimated for all classes (class varying). The class-varying approach led to convergence problems, even when only the intercepts were allowed to vary within classes. In contrast, the invariant approach models all provided admissible solutions, and hence this approach was adopted for all models. For anxiety models with two or more classes, the variance of the slope and quadratic parameters was small and non-significant and was therefore set to zero. A two-class solution was optimal for the depression, anxiety and post-traumatic stress models. Multiple fit indices were considered when deciding on the optimal number of classes; these included Bayesian information criterion, Akaike information criterion, sample-adjusted Bayesian information criterion, entropy and average posterior probabilities, the Lo-Mendell-Rubin likelihood ratio test and the bootstrapped likelihood ratio test.^25–28^ The size of the smallest class was also taken into account as per recommendations by Berlin et al,^29^ and plots were inspected to check that the class trajectories were clearly differentiated. The three-step manual approach was used to incorporate predictors into the models.^30^ This involved using the logits for the classification probabilities for most likely latent class membership (uncertainty rates) to fix class membership in the conditional models, thereby preventing class membership from being influenced by the inclusion of the covariates in the models. The predictors were entered into the models in three blocks: personal and demographic factors, organisational factors and support factors. Multiple imputation was used to deal with missing data on the covariates.

## Results

A two-class model provided the best fit for the depression, anxiety and post-traumatic stress symptom scores. See Supplementary Materials for further details.

### Trajectories for the two-class depression, anxiety and post-traumatic stress unconditional growth mixture models

**Fig. 1:**
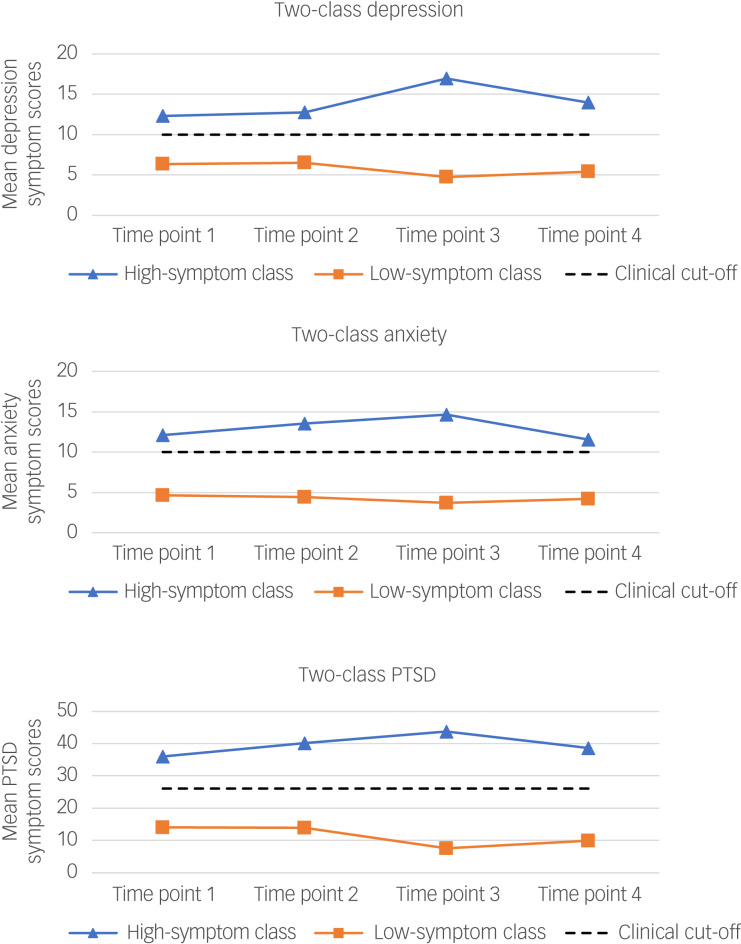
Trajectories for the two-class depression, anxiety and PTSD unconditional growth mixture models. PTSD, post-traumatic stress disorder.

In the depression, anxiety and post-traumatic stress two-class unconditional growth mixture models, a low-symptom class and a high-symptom class emerged. Means weighted by estimated class probabilities are shown in Fig. 1 for the two-class depression, anxiety and PTSD growth mixture models. The low-symptom class represented the largest proportion of the sample in the depression (*n* = 510, 87%), anxiety (*n* = 495, 85%) and PTSD (*n* = 492, 84%) models. Mean scores for the low-symptom class were at their highest at time points 1 and 2, dropped down at time point 3 and then rose again at time point 4. In the depression, anxiety and post-traumatic stress models, mean symptom scores remained well below the clinical cut-off point throughout the pandemic for the low-symptom class. In contrast, the high-symptom class represented a much smaller proportion of health and social care staff for the depression (*n* = 75, 13%), anxiety (*n* = 90, 15%) and PTSD (*n* = 93, 16%) models. For all three models, throughout the peaks and troughs of the pandemic, the high-symptom class reported levels in excess of clinical cut-offs (see Fig. 1). This class is very distinct from the low-symptom class in that levels of distress actually continued to rise until time point 3, before finally dropping off at time point 4.

Mean growth parameters scores for the depression, anxiety and post-traumatic stress unconditional growth mixture models are shown in Table 2. For depression and PTSD, as the models were specified to measure change from time points 2 to 3, the slope parameters indicate that although levels increased from time points 2 to 3 for the high-symptom class, levels actually fell for the low-symptom class. The slope and quadratic parameters in the anxiety model reflect the curved trajectories of the high- and low-symptom classes. Anxiety scores for the high-symptom class increased up until time point 3, then dropped off at time point 4. By contrast, anxiety scores for the low-symptom class show a downward trend between time points 1 and 3, which then increased as COVID-19 hospital admissions started to rise again in Northern Ireland.

**Table 2:** Mean scores for the growth parameters in the two-class unconditional models

Growth parameter	Depression		Anxiety		Post-traumatic stress disorder	
	High-symptom class	Low-symptom class	High-symptom class	Low-symptom class	High-symptom class	Low-symptom class
	Mean (s.e.)	Mean (s.e.)	Mean (s.e.)	Mean (s.e.)	Mean (s.e.)	Mean (s.e.)
Intercept	12.45 (0.80)***	6.43 (0.27)***	11.67 (0.86)***	4.86 (0.33)***	37.46 (1.56)***	13.37 (0.91)***
Slope	4.26 (0.75)***	−1.67 (0.20)***	3.76 (1.34)**	−0.78 (0.30)**	5.80 (1.91)**	−5.89 (0.73)***
Quadratic	−	−	−1.23 (0.39)**	0.17 (0.08)*	−	−

* *P* < 0.05, **P < 0.01, ***P < 0.001.

### Prediction of class membership

Table 3 shows the logistic coefficients for the models predicting depression, anxiety and post-traumatic stress class membership. The coefficients represent the log odds of being in the high-symptom class versus the low-symptom class. In block 1, age, occupation, gender, exposure to COVID-19 and having at least one COVID-19 risk factor were entered. Being younger was associated with greater likelihood of being in the high-symptom class than the low-symptom class for the depression and anxiety models. Having a higher level of exposure to COVID-19 was associated with greater likelihood of belonging to the high-symptom class in the anxiety and post-traumatic stress models. Subsequently, in block 2, whether workers managed patients with COVID-19, their perceived effectiveness of communication regarding COVID-19 and if they were asked to consider being redeployed were entered into the models. Following entry of these organisational factors, in all three models the high-symptom class perceived communication from their organisation to be less effective than those from the low-symptom class. In the post-traumatic stress model, those who managed patients with COVID-19 were more likely to be in the high-symptom class. The predictors identified as significant at block 1 generally remained so at block 2, except for the age predictor in the depression model. Although occupation had no association with class in the post-traumatic stress model after block 1 entry, results for block 2 indicated that administrative and clerical staff were more likely to be in the high-symptom class than nursing and midwifery staff. Finally, in block three, the following variables were entered: if team supports were available, if staff well-being supports were used, the number of team supports available and the number of staff well-being supports used. Block 1 and 2 predictors were largely stable following block 3 entry, with the exception of managing patients with COVID-19 in the PTSD model. None of the support variables were related to class membership in the depression model. In both the anxiety and post-traumatic stress models, members of the high-symptom class tended to report having fewer team supports available to them than the low-symptom class. In the PTSD model, those in the high-symptom class reported using a significantly greater number of supports than those in the low-symptom class.

**Table 3:** Logistic regression of predictors of class membership (*N* = 585)

	Estimate (s.e.)								
	Depression			Anxiety			Post-traumatic stress disorder		
	Block 1	Block 2	Block 3	Block 1	Block 2	Block 3	Block 1	Block 2	Block 3
Occupation (nursing and midwifery as reference)									
Administrative and clerical	0.58 (0.48)	0.93 (0.57)	0.88 (0.60)	0.41 (0.43)	0.87 (0.55)	0.72 (0.57)	0.38 (0.37)	0.98 (0.42)*	0.91 (0.44)*
Medical	−0.05 (0.84)	−0.17 (0.91)	−0.06 (0.90)	−0.19 (0.85)	−0.26 (0.89)	−0.02 (0.83)	−0.07 (0.61)	−0.31 (0.65)	−0.14 (0.68)
Professional and technical	0.02 (0.54)	0.20 (0.57)	0.19 (0.60)	−0.43 (0.49)	−0.21 (0.54)	−0.29 (0.56)	−0.50 (0.43)	−0.27 (0.43)	−0.25 (0.45)
Social services	0.18 (0.61)	0.35 (0.68)	0.37 (0.68)	−0.40 (0.60)	−0.17 (0.69)	−0.25 (0.64)	−0.55 (0.59)	−0.25 (0.64)	−0.20 (0.61)
Other	0.95 (0.71)	1.03 (0.72)	1.01 (0.73)	0.02 (0.75)	0.04 (0.81)	−0.20 (0.85)	0.20 (0.62)	0.41 (0.63)	0.32 (0.66)
Gender (male as reference)									
Female	0.52 (0.52)	0.60 (0.55)	0.62 (0.57)	1.06 (0.57)	1.14 (0.63)	1.15 (0.61)	0.09 (0.35)	0.13 (0.38)	−0.15 (0.39)
Age, years	−0.03 (0.01)*	−0.03 (0.02)	−0.03 (0.02)	−0.05 (0.01)***	−0.05 (0.01)**	−0.04 (0.01)**	−0.00 (0.01)	0.00 (0.01)	0.01 (0.01)
Exposure to COVID-19	0.17 (0.11)	0.14 (0.12)	0.15 (0.12)	0.25 (0.10)*	0.22 (0.10)*	0.23 (0.11)*	0.23 (0.10)*	0.21 (0.10)*	0.21 (0.10)*
Have at least one COVID-19 risk factor	0.61 (0.35)	0.61 (0.38)	0.60 (0.38)	0.36 (0.35)	0.37 (0.37)	0.35 (0.38)	0.58 (0.30)	0.56 (0.32)	0.55 (0.33)
Managed patients with COVID-19		0.28 (0.46)	0.26 (0.47)		0.39 (0.43)	0.37 (0.45)		0.77 (0.37)*	0.76 (0.39)
Perceived effectiveness of communication regarding COVID-19		−0.53 (0.15)***	−0.54 (0.17)**		−0.49 (0.14)***	−0.46 (0.15)**		−0.45 (0.12)***	−0.45 (0.14)**
If they were asked to consider being redeployed		−0.16 (0.40)	−0.11 (0.42)		−0.02 (0.34)	0.05 (0.35)		−0.04 (0.32)	0.01 (0.34)
If team supports were available			0.41 (0.53)			0.62 (0.48)			0.58 (0.44)
If they used staff well-being supports			−0.59 (0.59)			−0.36 (0.72)			−0.63 (0.50)
Number of team support available			−0.25 (0.30)			−0.60 (0.24)*			−0.50 (0.24)*
Number of staff well-being supports used			0.39 (0.26)			0.53 (0.42)			0.60 (0.23)**

Block 1: personal and demographics factors; block 2: organisational factors; block 3: supports.

* *P* < 0.05, **P < 0.01, ***P < 0.001.

## Discussion

This study examined mental health symptoms (depression, anxiety and PTSD) of healthcare staff over four time points during the COVID-19 pandemic. Applying growth mixture modelling, two subpopulations were identified within the workforce that differed in terms of how the pandemic affected their long-term mental health, as reflected by their qualitatively distinct mental health symptom trajectories. In the depression, anxiety and post-traumatic stress models, two different classes emerged: a group with consistently mild/no symptoms, called the low-symptom class; and a group with persistent moderate/severe symptoms, called the high-symptom class. The low-symptom class represented the largest proportion of the sample in all models (84–87%), with symptom scores mirroring the pattern of COVID-19 hospital admissions during the period examined.^18^ By contrast, the high-symptom class represented a smaller proportion of health and social care staff for all models (13–16%). The symptom levels of the high-symptom class continued to rise throughout the quieter period of hospital activity in Northern Ireland, with the drop-off in levels being delayed by 3 months relative to that observed for the low-symptom class.

Several cross-sectional studies have been published highlighting the impact of the COVID-19 pandemic on the mental health and well-being of healthcare staff.^7^ A number of studies have reported on two waves of longitudinal data.^9,31^ The present study, to the best of our knowledge, is the first to longitudinally examine the mental health of subpopulations of healthcare staff across several waves of the COVID-19 pandemic. Although as many as a third of healthcare workers have been estimated to have moderate-to-severe symptoms at a single point in time during early phases and significant peaks of the COVID-19 pandemic,^9^ the growth mixture modelling approach employed here demonstrates that throughout a 9-month period covering both busy and quieter periods of COVID-19 hospital activity, the number with persistently high symptoms is lower, but still substantive (13–16%).

Similar to the general population research by McPherson et al,^16^ we found evidence of a group of healthcare workers with persistently high symptoms, and that the vast majority of healthcare staff have low stable symptoms. In contrast to McPherson et al,^16^ we did not find any evidence of an ‘improving group’ who struggle at the start and then improve. Possibly, the differences in ‘population’ and ‘time’ may explain this. We completed our study further on in the pandemic, and perhaps mental health difficulties are more persistent at that point, i.e. those who are likely to adapt have already done so long before the second and third wave. Healthcare staff may well also be at greater risk of persistent difficulties than the wider population because of the high levels of stressors they experience, particularly an increased dose effect of ‘organisational traumas’ (e.g. exposure to death and dying) in addition to generic aversive events during the pandemic. Indeed, the cumulative, complex and long-term exposure to multiple forms of healthcare work-related traumatic experiences is likely to be linked to more enduring mental health problems.^12–14^

The low-symptom class showed a decrease in symptoms during quieter periods of COVID-19-related hospital activity, yet the reverse was true for the high-symptom class. There is a lack of research investigating factors influencing the course of mental health problems in healthcare staff, but, speculatively, it may be the case that the quieter periods allowed the high-symptom class to begin to focus on and process their difficult experiences. Indeed, delayed consequences of trauma experiences have been reported, and multiple variations of the longitudinal course of mental health problems, including trauma reactions, are common.^32,33^

### Implications

Previous research has highlighted the high levels of mental health symptoms during the pandemic, and supported calls for the provision of staff support on individual, team and systemic organisation levels.^6,7,9,34–36^ The present study focused on a previously neglected organisational research area, the impact of pandemic support frameworks on healthcare staff's mental health.^37^ Access to timely, individual mental health treatments has been argued to be particularly important in terms of treating emerging psychological problems.^38^ However, the present study reveals that staff with specific presentations and symptom intensity may require different levels and types of support. For example, individuals with high-symptom PTSD may need both a greater amount of support and a set of specific types of support that were not available during the pandemic. Such an interpretation comes from the findings that this subgroup used more well-being interventions but also reported that not enough supports were available to them. In contrast, the vast majority of staff that fell into the low-symptom class, with modest ups and downs in levels of symptoms, may best be managed by generic organisational changes, including a reduction in health system pressures and low-intensity staff well-being initiatives.^39^

Regarding those with more severe and persistent difficulties, age was a relevant factor, with younger staff at risk of greater anxiety and, to a lesser extent, depression, but not PTSD. In these analyses, age could be an artefact of staff experience level, which has been shown to be a protective factor against mood-related reactions associated with COVID-19 healthcare duties (e.g. job dissatisfaction).^39^ In contrast, risk of PTSD was less affected by staff demographics and, understandably, more affected by the level of exposure to potentially traumatic work experiences (e.g. managing patients with COVID-19) and availability of support. These and other findings suggest there may be a need to increase the accessibility or awareness of team supports. Specifically, in both the anxiety and post-traumatic stress models, members of the high-symptom class tended to report having fewer team supports available to them than the low-symptom class. However, in the PTSD model, those in the high-symptom class reported using a significantly greater number of supports than those in the low-symptom class.

In all three models, the high-symptom class perceived communication from their organisation to be less effective than those from the low-symptom class. This finding is in keeping with previous literature demonstrating the importance of organisational factors such as communication from healthcare organisations during COVID-19, but also more generally, in terms of staff well-being.^9,15^ Effective communication is essential during COVID-19, as the work environment created by a pandemic is an unknown to most staff and practice guidance can change daily. There were, of course, massive challenges to organisations and their ability to communicate during the pandemic. These included a big increase in the flow of information and a great deal of uncertainty and fear.^40^ It is therefore unsurprising that there have been many calls for frequent, clear, simple and transparent communication to all health service staff.^41^ Successful recovery of the health system will involve management and leadership that value staff mental health and well-being.^1^

### Limitations

It must be noted that this paper is based on four time points during the COVID-19 pandemic. Only one time point fell within a quiet period of COVID-19 hospital activity. Although some of the impact of the pandemic seems to be waning, hospital admissions remain high and there remains persistent stress on the healthcare system. Hence, there is a need for a longer span of data covering the post-COVID-19 period, so that we can study the ‘recovery’ phase. Conditions such as depression and PTSD can have a long and complex course.^42,43^ It may well be that some staff take longer to recover from the stress of this time and will only do so when the pandemic recedes. In addition, the current study only examined a circumscribed pool of predictor variables and did not differentiate between primary and secondary stressors, the latter of which has been reported as having greater effect.^44^ There is also the possibility that well-documented risk factors shown to have contributed to poor mental health outcomes in the general public during COVID-19 could also be relevant to the high-symptom class (e.g. diet, social isolation).^45^ These variables were not measured in the present investigation and should be examined in future studies, along with staff pre-existing mental health history. Some of the participants in the high-symptom class may have had psychological difficulties before the pandemic, and the persistence of these mental health problems may be contingent on generic COVID-19-related experiences as well as healthcare vocational experiences.

Unfortunately, the strict infection control rules in place during the pandemic made it difficult to reach staff in non-desk-based occupations and front-line roles (e.g. nursing and midwifery); less consistent participation from staff in those sectors led to staff in such roles being slightly underrepresented in the longitudinal sample. In addition, the findings are based on self-report questionnaires and not clinical interviews with staff, hence the results are indicative of ‘probable’ clinical levels of mental health difficulties rather than actual caseness. However, although clinical interviews remain the gold standard of diagnostic assessment, there are often moderate-to-good levels of agreement between these two methods.^46^

In conclusion, this is the first study to report on a group of healthcare staff that have persistent difficulties with mental health symptoms over four time points during the COVID-19 pandemic. It absolutely underlines the need to provide comprehensive staff support services to maintain and sustain a safe working environment in healthcare. As part of a comprehensive response to staff well-being, support services will need a strategy to target those with persistent problems who may be taking longer to recover from the experience of the pandemic. It also may be important to target specific groups, such as younger staff and those working in administrative roles. The study also highlights the importance of communication in healthcare organisations, and suggests this may be an importance target in relation to staff well-being.

## Data Availability

The data that support the findings of this study are available from the corresponding author, C.S., upon reasonable request.

## Supplementary material

For supplementary material accompanying this paper visit http://doi.org/10.1192/bjo.2023.497. click here to view supplementary material
