# Supplementary material for: Healthcare staff mental health trajectories during the COVID-19 pandemic: findings from the COVID-19 Staff Wellbeing Survey
Source: BJPsych Open. 2023 Jun 22;9(4):e112. doi: 10.1192/bjo.2023.497 (PMC10305016; doi:10.1192/bjo.2023.497)
Supplement: Supplementary file 1 [file S2056472423004970sup001.docx]

**Supplementary materials**

Table 1. Demographic profiles of the Staff Wellbeing survey from Time 1-4.

| Variable | Statistics | Score | | | |
| --- | --- | --- | --- | --- | --- |
|  |  | Time 1 (n=3,834) | Time 2 (2,898) | Time 3 (n=2,480) | Time 4 (n=2,119) |
| Age | Mean (SD) | 43.61 (10.54) | 43.87 (10.68) | 45.18 (10.31) | 45.04 (10.40) |
| Gender |  |  |  |  |  |
| Male | Frequency (%) | 664 (17%) | 486 (17%) | 411 (17%)  2067 (83%) | 374 (18%) |
| Female | Frequency (%) | 3161 (83%) | 2409 (83%) |  | 1744 (82%) |
| Occupation |  |  |  |  |  |
| Nursing & Midwifery | Frequency (%) | 903 (24%) | 695 (24%) | 575 (23%) | 499 (24%) |
| Administrative & Clerical | Frequency (%) | 1059 (28%) | 813 (28%) | 746 (30%) | 586 (28%) |
| Medical | Frequency (%) | 243 (6%) | 175 (6%) | 128 (5%) | 114 (5%) |
| Professional & Technical | Frequency (%) | 771 (20%) | 618 (21%) | 538 (22%) | 496 (23%) |
| Social Services | Frequency (%) | 548 (14%) | 348 (12%) | 310 (13%) | 257 (12%) |
| Other | Frequency (%) | 310 (8%) | 249 (9%) | 183 (7%) | 167 (8%) |

Unconditional Growth Curve models

Unconditional growth curve models were fit to the psychological wellbeing data for times 1-4. The fit indices revealed that a quadratic model with the slope variance constrained to zero provided the best fit for the anxiety model, and a free time score approach was more appropriate for the depression, PTSD, and insomnia models. Fit indices for the selected unconditional growth curve models are shown in Supplementary Table 1. All models showed acceptable fit with fit indices falling within acceptable ranges (Hu & Bentler, 1999); specifically, SRMR < .08; RMSEA < .06; CFI and TLI > .95. In the models adopting a free time scores approach, Time 2 was coded as 0, as this was when the pandemic peaked in Northern Ireland (NI) and visual analysis indicated this was also when depression, post-traumatic stress and insomnia levels were at their highest (see Supplementary Table 2). For all models, Time 3 was coded as 1, reflecting the point when both the sample means and NI COVID-19 hospitalisations were at their lowest (Department of Health, 2021). The coding metric applied to each of the free time scores models allows the slope in each model to be interpreted as the difference between when psychological wellbeing difficulties were at their highest and lowest during the COVID-19 pandemic.

Supplementary Table 1: Fit statistics for the unconditional psychological wellbeing growth curve models (N=585)

|  | Depression | Anxiety | PTSD | Insomnia |
| --- | --- | --- | --- | --- |
| *x*^2^ (df) | 4.07 (2), *p*=.131 | 6.51 (4), *p*=.165 | 2.00 (2), *p* = .367 | 6.34 (3), *p*=.096 |
| CFI | 1.00 | 1.00 | 1.00 | 1.00 |
| TLI | .99 | 1.00 | 1.00 | 1.00 |
| RMSEA | .04 | .03 | .00 | .04 |
| SRMR | .02 | .03 | .02 | .03 |

Supplementary Table 2: Means and standard deviations on the psychological wellbeing measure across time

|  | Mean (SD) | | | |
| --- | --- | --- | --- | --- |
|  | Time 1( n=457) | Time 2 (n=526) | Time 3 (n=514) | Time 4 (n=427) |
| Depression | 7.22 (5.75) | 7.39 (5.68) | 6.51 (5.65) | 6.59 (5.41) |
| Anxiety | 5.96 (5.27) | 5.91 (5.00) | 5.42 (5.15) | 5.40 (4.79) |
| PTSD | 17.94 (16.40) | 18.23 (16.92) | 13.42 (15.91) | 14.55 (16.33) |
| Insomnia | 9.97 (5.81) | 10.22 (5.94) | 9.58 (5.89) | 9.72 (5.98) |

Significant variation in growth curve parameters (e.g. intercept, slope) can indicate that sub-populations exist within the sample (Kandauda et al., 2016). Supplementary Table 3 shows that there was significant variation in intercept for all models and significant variation in slope for the depression, and post-traumatic stress models, and in the quadratic component of the anxiety model.

Supplementary Table 3: Unconditional growth curve models for the psychological wellbeing measures (N=585)

|  | Depression | Anxiety | PTSD | Insomnia |
| --- | --- | --- | --- | --- |
| Intercept | 7.39 (0.24);  *p* < .001 | 5.94 (.229);  *p* < .001 | 18.18 (0.71);  *p* < .001 | 10.20 (0.26);  *p* <. 001 |
| Slope | -0.85 (0.19);  *p* < .001 | -0.00 (0.21);  *p* = .996 | -4.61 (0.56);  *p* < .001 | -0.42 (0.20);  *p*=.038 |
| Quadratic | - | -0.07 (0.07);  *p* = .276 | - | - |
| Variance (intercept) | 23.71 (2.15);  *p* < .001 | 19.17 (1.42);  *p* < .001 | 189.48 (16.26);  *p* < .001 | 29.21 (3.041);  *p* <.001 |
| Variance (slope) | 4.91 (1.95);  *p*=.012 | - | 64.58 (21.00);  *p* = .002 | 0.26 (2.05);  *p* = .899 |
| Variance (quadratic) | - | .07 (.03);  *p* = .012 | - | - |
| R* | -3.02 (1.66);  *p* = .068 | -.34 (.13);  *P* = .007 | -33.43 (14.37);  *p* = .020 | -1.97 (2.15);  *p* = .361 |

* intercept with slope covariance for depression, PTSD and Insomnia models. Intercept with quadratic covariance for anxiety model.

Unconditional growth mixture models

In the next stage of the analysis growth mixture modelling was used to explore the significant variation in slope/quadratic parameters revealed in the depression, anxiety and post-traumatic stress growth curve models, with a view to identifying any sub-populations that may exist. The fit indices for the unconditional growth mixture models for 1-5 classes are shown in Supplementary Table 4. While AIC, BIC, and SABIC continued to decrease throughout 1 to 5 classes, scree plots revealed (Nylund-Gibson & Choi, 2018) that the largest decrement occurred between 1 and 2 classes for the depression, anxiety and post-traumatic stress models. Inspection of scree plots showed that for the depression and post-traumatic stress models the reduction between 1 and 2 classes was markedly greater than the decrements between the other sequential class numbers examined. Entropy was highest for the 2-class solution of the depression and post-traumatic stress models, and for the 4- and 5-class solutions of the anxiety models. For all models LMR-LRT suggested that a 2-class solution provided a better fit than a 1-class solution, and a 3-class solution did not provide an improvement in fit over a 2-class solution. BS-LRT values continued to suggest an improvement in model fit as additional classes were added to the models. The only models for which the average latent class probabilities were within acceptable limits (.80 and above; Weller et al, 2020) were the 2-class depression and anxiety models, and the 2- and 3-class post-traumatic stress models. Inspection of the plots in conjunction with the average latent class probabilities highlighted that when moving from a 2-class solution, to a 3-class solution, the emerging additional class had an unacceptable (<.80) average latent class probability for the anxiety and depression models. On balance the fit indices largely supported a 2-class solution for the depression, anxiety and post-traumatic stress models.

Supplementary Table 4: Fit indices for unconditional growth mixture models with 1- to 5-classes (N=585).

|  | Fit indices | 1-class | 2-class | 3-class | 4-class | 5-class |
| --- | --- | --- | --- | --- | --- | --- |
| Depression | AIC | 11068.85 | 10921.27 | 10882.14 | 10822.92 | 10807.67 |
|  | BIC | 11121.31 | 10986.84 | 10960.83 | 10905.98 | 10903.85 |
|  | SABIC | 11083.21 | 10939.22 | 10903.69 | 10845.66 | 10834.00 |
|  | Entropy | - | .88 | .83 | .85 | .82 |
|  | LMR-LRT *p* value | - | <.001 | .564 | .119 | .486 |
|  | BS-LRT *p*-value | - | <.001 | <.001 | <.001 | <.001 |
| Anxiety | AIC | 10697.92 | 10588.14 | 10497.62 | 10412.97 | 10365.45 |
|  | BIC | 10741.64 | 10640.60 | 10567.56 | 10500.40 | 10470.37 |
|  | SABIC | 10709.89 | 10602.50 | 10516.77 | 10436.91 | 10394.18 |
|  | Entropy | - | .85 | .86 | .89 | .87 |
|  | LMR-LRT | - | .015 | .268 | .011 | .205 |
|  | BS-LRT | - | <.001 | <.001 | <.001 | <.001 |
| PTSD | AIC | 15293.40 | 15071.78 | 14969.38 | 14903.11 | 14887.15 |
|  | BIC | 15345.86 | 15137.36 | 15048.07 | 14994.92 | 14992.06 |
|  | SABIC | 15307.77 | 15089.74 | 14990.93 | 14928.25 | 14915.87 |
|  | Entropy | - | .91 | .89 | .88 | .86 |
|  | LMR-LRT | - | <.001 | .085 | .002 | .414 |
|  | BS-LRT | - | <.001 | <.001 | <.001 | <.001 |

Multiple imputation

Multiple imputation was used to handle missing data on the Time 1 predictor variables. The multiple imputation was run in Mplus using the Markov Chain Monte Carlo method to generate 10 datasets. The data on the Time 1 predictor variables were assumed to be missing at random as variables at other time points were predictive of missingess on the Time 1 variables. The outcome variables were included in the imputation procedure as well as the Time 1-4 versions of the predictor variables – this included variables that were predictive of missingness at Time 1. The final logistic regression models to predict class membership were run with complete cases to allow comparison with those based on multiple imputation (Supplementary Table 5). Broadly speaking, the pattern of results was similar for the complete case and multiple imputation analyses. Given that the data are assumed to be missing at random, the minor discrepancies observed are likely to reflect the use of appropriate predictors of missingness to create the multiple imputation datasets. While Multiple Imputation is considered to be one of the gold standard approaches for dealing with missing data (e.g. Erler, Rizopoules, Rosmalen, Jaddoe, & Franco, 2016), the assumption that data are missing at random is a disadvantage of the approach. While predictors of missingness were included in the multiple imputation model, it is possible that other variables not included within the present study also predict missingness.

Supplementary Table 5: Model results for complete cases and those based on multiple imputation (Block 3)

|  |  | Estimate (SE) | | | | | |
| --- | --- | --- | --- | --- | --- | --- | --- |
|  |  | Depression | | Anxiety | | PTSD | |
|  |  | Complete cases (n=456) | Multiple Imputation (n=585) | Complete cases (n=456) | Multiple Imputation (n=585) | Complete cases (n=456) | Multiple Imputation (n=585) |
| Occupation | Nursing & Midwifery (reference) |  |  |  |  |  |  |
|  | Administrative and Clerical | 1.07 (.65) | .88 (.60) | .64 (.62) | .72 (.57) | .72 (.46) | .91 (.44)* |
|  | Medical | -.16 (1.21) | -.06 (.90) | .03 (.86) | -.02 (.83) | -.44 (.69) | -.14 (.68) |
|  | Professional & technical | .42 (.67) | .19 (.60) | -.34 (.61) | -.29 (.56) | -.22 (.47) | -.25 (.45) |
|  | Social services | .78 (.73) | .37 (.68) | -.27 (.67) | -.25 (.64) | -.28 (.60) | -.20 (.61) |
|  | Other | 2.26 (.80)** | 1.01 (.73) | .37 (.98) | -.20 (.85) | .93 (.69) | .32 (.66) |
| Gender | Male (reference) |  |  |  |  |  |  |
|  | Female | 1.32 (.99) | .62 (.57) | 1.27 (.77) | 1.15 (.61) | .13 (.43) | -15 (.39) |
| Age (years) | | -.03 (.02) | -.03 (.02) | -.04 (.02)* | -.04 (.01)** | .02 (.02) | .01 (.01) |
| Exposure to COVID-19 | | .17 (.16) | .15 (.12) | .27 (.12)* | .23 (.11)* | .24 (.11)* | .21 (.10)* |
| Have at least one COVID-19 risk factor | | .97 (.47)* | .60 (.38) | .50 (.43) | .35 (.38) | .45 (.35) | .55 (.33) |
| Managed patients with COVID-19 | | .02 (.47) | .26 (.47) | .11 (.49) | .37 (.45) | .66 (.41) | .76 (.39) |
| Perceived effectiveness of communication regarding COVID-19 | | -.63 (.19)** | -.54 (.17)** | -.54 (.17)** | -.46 (.15)** | -.45 (.15)** | -.45 (.14)** |
| If asked to consider being redeployed | | -.11 (.44) | -.11 (.42) | .08 (.37) | .05 (.35) | -.01 (.34) | .01 (.34) |
| If team supports were available | | .36 (.59) | .41 (.53) | .71 (.53) | .62 (.48) | .55 (.47) | .58 (.44) |
| If used staff wellbeing supports | | -.86 (.69) | -.59 (.59) | -.52 (.71) | -.36 (.72) | -.57 (.53) | -.63 (.50) |
| Number of team support available | | -.21 (.33) | -.25 (.30) | -.65 (.27)* | -.60 (.24)* | -.52 (.24)* | -.50 (.24)* |
| Number of staff wellbeing supports used | | .51 (.30) | .39 (.26) | .69 (.40) | .53 (.42) | .61 (.24)** | .60 (.23)** |

**p* < .05; ***p* < .01; ****p* < .001; Block 1 = Personal and demographics factors; Block 2 = Organisational factors; Block 3 = Supports
